# Prevalence of somatic and urogenital symptoms as well as psychological health in women aged 45 to 55 attending primary health care: a cross-sectional study

**DOI:** 10.1186/s12905-017-0480-1

**Published:** 2017-12-08

**Authors:** Lena Rindner, Gunilla Strömme, Lena Nordeman, Margareta Wigren, Dominique Hange, Ronny Gunnarsson, Gun Rembeck

**Affiliations:** 1 Närhälsan, Skene Health Care Center, Varbergsvägen 80, SE-511 81 Skene, Sweden; 2 Närhälsan Svenljunga Antenatal Clinic, Svenljunga, Sweden; 3 Research and Development Center Södra Älvsborg, Närhälsan, Research and Development, Primary Health Care Region, Västra Götaland, Sweden; 4 Department of Health and Rehabilitation, Unit of Physiotherapy, University of Gothenburg, Sahlgrenska Academy, Institute of Neuroscience, Physiology, Gothenburg, Sweden; 5 Närhälsan, Svenljunga Health Care Center, Svenljunga, Sweden; 6 Department of Public Health and Community Medicine/Primary Health Care, Institute of Medicine, the Sahlgrenska Academy, University of Gothenburg, Gothenburg, Sweden; 7 General Practice and Rural Medicine, Cairns Clinical School, College of Medicine and Dentistry, James Cook University, Townsville, Australia; 8 Närhälsan Borås Adolescent Health Centre, Kvarngatan 4, 50336 Borås, Sweden

**Keywords:** Menopause, Cardiovascular diseases, Sleep, Stress, Women’s health, Sexuality, Mental health

## Abstract

**Background:**

Women’s physical and mental ill-health such as stress-related symptoms, depression, pain, hypertension and urogenital health shows a marked increase around the ages 45–55 years. These women are an important group for Primary Health Care (PHC) due to their prevalent symptoms and illnesses. The aim of this study was to estimate the prevalence of somatic, psychological and urogenital symptoms in women aged 45–55 attending PHC and evaluate factors associated with severe symptoms.

**Methods:**

One hundred and thirty-one women were recruited from PHC in southwestern Sweden. Data were obtained from two self-reported questionnaires, the Menopause Rating Scale (MRS) and the Montgomery-Asberg Depression Rating Scale (MADRS).

**Results:**

Exhaustion, depressive mood, muscle and joint problems, sleep and sexual problems were the most prevalent reported symptoms. Half of the women reported heart discomfort. Depression and increasing age were correlated to more severe symptoms.

**Conclusion:**

We recommend that cardiovascular risk factors, musculoskeletal symptoms, sexual problems, sleeping problems and mental health should be actively asked for when women aged 45 to 55 attend PHC. We propose that preventive counselling of women in PHC before the age 45 should be evaluated in future studies.

## Background

The transition between the age 45–55 is for many women a troublesome time of life with decreased oestrogen levels characterized by ovarian aging and gradual loss of ovarian function [1]. Decreased oestrogen levels may affect the cardiovascular system, cause skeletal, joint and muscle problems, as well as vasomotor and urogenital symptoms [2–4].

The debut of menopause varies between different countries from age 47 to 51 [1, 5]. The mean age for entering menopause in Sweden is 51 [6]. The most frequently occurring symptoms among women aged 45–55 years are pain, sleep disorders, physical and mental fatigue, depression and sexual problems. Hot flushes may also be seen [5, 7–10].

Many symptoms such as mental exhaustion, sleep disorders and the metabolic syndrome, independent risk factors of developing cardiovascular disease, are increasing during menopause [9, 11]. Women’s lipid profiles change for the worse, leading to increased arteriosclerosis and coronary artery disease [3, 4, 9, 12]. Women over 50 have a 46% risk for coronary artery disease at some point during the remainder of their lives [3, 12, 13]. These symptoms and conditions occurring in the age group 45–55 years are not necessarily correlated to oestrogen levels and menopause [1].

Mental illness, particularly depressive symptoms, also shows a marked increase during this phase of life [14, 15] and changes in reproductive hormone dynamics during the menopausal transition may contribute to the risk for depression [16]. This phase of life has been labelled “the window of vulnerability” [14].

### Middle aged unwell women in primary health care

Symptoms such as malaise, attention deficit, irritability, physical and psychological fatigue are more common today in all age groups, particularly in women. Poor mental health has now become the most common cause of sick leave with the greatest increase for women “in the prime of life” [17, 18]. It is apparent that women suffer long term sickness and ill health more than men [19, 20]. Hence, women in the age group 45–55 years are a very important target group for primary health care (PHC).

PHC is the basis of health care delivery in Sweden and is responsible for the basic needs of the population for healthcare, care of major public health diseases and primary prevention. It has the responsibility for the individual’s overall conditions and needs [21].

The district nurse (DN), also commonly labelled Advanced Practice Nurse (APN) in Primary Health Care with a multidisciplinary PHC background, together with midwifes plays an important and autonomous role in the Swedish PHC system [22]. In Swedish PHC, DN and midwives meet women aged 45–55 years with concerns about their bodily changes and psychological symptoms [23]. Many of these issues involve the normal changes of this phase of life, but cause much concern and increased perception of illness for some women [24]. Prevention based on a holistic approach leads to better care for women with these symptoms [25, 26].

Increased knowledge can improve existing primary care routines for these women. Despite a few studies [8, 27] there is still a lack of knowledge of the prevalence of somatic, psychological and urogenital symptoms as measured by the Menopause Rating Scale (MRS) and the Montgomery-Asberg Depression Rating Scale (MADRS) in PHC. The aim of this study was to estimate the prevalence of these symptoms in women attending PHC and evaluate factors associated with more severe symptoms.

## Methods

This cross sectional study was done from March 2009 until December 2010. The study was approved by the Regional Ethical Review Board in Gothenburg Sweden (registration number 041–09; T503–14). Informed consent was obtained from all participants and confidentiality was ensured. All participants were given a description of the study, and informed about the right to decline participation or to withdraw from participation.

Women aged 45–55 years that, for any reason, visited the PHC centers in two municipalities in southwestern Sweden and in a certain predefined period of time were consecutively asked for participation. The inclusion criteria were women aged 45–55 years with no impaired understanding of the Swedish language. No limitation was imposed if they were using any kind of prescription drug.

## Data collection

Data collection for this study was collected by means of two self-reported questionnaires, The Menopause Rating Scale (MRS) and the Montgomery-Asberg Depression Rating Scale (MADRS). Apart from the two self-reported questionnaires, the women also answered demographic questions regarding age, educational level, family and work status.

### Menopause rating scale (MRS)

For evaluation of the prevalence and severity of symptoms possibly related to the menopaue, as developed by Heinemann and validated in Sweden, was used [28, 29]. The Menopause Rating Scale (MRS) was developed in the early 1990s to measure severity of symptoms and their impact on quality of life on aging women. MRS is validated and was initially published in German. The first translation was from German to English followed by translation to Swedish. The MRS scale is used worldwide and translated into several languages [29]. The MRS is a self-administrated questionnaire consisting of eleven items, divided into three subscales reflecting; somatic symptoms - hot flushes, coronary discomfort, sleeping problems and muscle and joint problems; psychological symptoms **-** depressive mood, irritability, anxiety and physical and mental exhaustion; and urogenital symptoms - sexual problems, bladder problems and vaginal dryness. Each item ranged from 0 (not present) to 4 (1 = mild; 2 = moderate; 3 = severe; 4 = very severe). The MRS total score is the sum of the scores obtained for each subscale. Values equal or above 9 (somatic), 7 (psychological), 4 (urogenital), and 17 (total) were used to define severe menopausal symptoms [28].

### Montgomery-Asberg depression rating scale (MADRS)

The Montgomery–Åsberg Depression Rating Scale (MADRS) was 1979 by British and Swedish researchers to measure the severity of depressive episodes in patients with mood disorders [30, 31]. MADRS is validated and used worldwide The MADRS consists of nine questions, each scored from 0 to 6, where higher score indicates more severe symptoms. The nine questions in MADRS include the symptoms; 1) Apparent Sadness 2) Inner Tension 3) Reduced Sleep 4) Reduced Appetite 5) Concentration Difficulties 6) Lassitude 7) Inability to Feel 8) Pessimistic Thoughts and 9) Suicidal Thoughts. The total MADRS score was interpreted as follows; 0–6 no depression, 7–19 mild depression, 20–34 moderate depression, >34 severe depression [32].

## Statistical analysis

Data are expressed as means and standard deviation (SD), median and percentiles and percentages. The MRS total score and somatic, urogenital and psychological subscale score were calculated separately. The MADRS was calculated for total score according to the international manual [32]. Women with no menstruation during the preceding 12 months were deemed as menopausal.

Prevalence of symptoms in MRS and prevalence of depressive symptoms in MADRS was described as numbers and proportions.

The MRS score was dichotomized by the mean value as follows; somatic symptoms ≥9 = 1/≤ 8 = 0, urogenital symptoms ≥4 = 1/≤ 3 = 0, psychological symptoms ≥7 = 1/≤ 6 = 0 and total score ≥ 17 = 1/≤ 16 = 0. A binary logistic regression was made for each dichotomized MRS score. Independent explanatory variables in the regression were: age 45–55 year, working status; work >1 h/w = 1/not working = 0, living with a partner = 1/no partner = 0, education tertiary school = 1/primary/secondary school = 0, depressive symptoms score measured with MADRS ≥7 = 1/< 6 = 0. Chi square test and Mann Whithey U test was used to compare baseline data between women in menopause and women still menstruating.

A number of unadjusted logistic regression was first done to assess the associations between each of the independent variables and the dependent variables of severe somatic, urogenital, psychological and total symptoms according to MRS*.* Significant variables were evaluated for zero order correlations. The next step was a forward stepwise multivariate (adjusted) logistic regression including the independent variables with a significant association to stronger symptoms according to MRS. The area under curve (AUC) was performed to validate any multivariate logistic regression models with at least two independent variables [33]*.* Finally, any multivariate logistic model was transformed into a probability nomogram predicting the probability of severe menopausal symptoms. The level of significance was set to *p* < 0.05. The SPSS Windows version 19.0 was used for statistical analyses.

## Results

One hundred thirty-one women accepted participation. Two women declined participation due to lack of time and nineteen failed to participate without giving a reason. Hence, 110 women aged 45 to 55 were assessed (Table 1). Information about menstruation was missing for 16 women due to 13 still used contraceptive treatments with hormones, one had a previous hysterectomy and two did not provide a clear statement on menstruation.

**Table 1:** Demography, depression and symptom scores among women 45–55 years

	Total score (110)		Pre/peri^a^ (69)		Post^b^ (25)		*P*-value
Age							
(y^)c^	50 (3)		50 (3.1)		52 (3.0)		**0.0049**
Education (y)^d^							
Primary school (< 9)	20 (18)		8 (12)		7 (28)		
Secondary school (10–12)	51 (46)		31 (45)		11(44)		**0.0065**
Tertiary school (>12)	39 (36)		30 (44)		7 (28)		
Work^d^							
In work	92 (84)		57 (83)		21 (84)		0.87
Family status^d^							
Living with a partner	98 (89)		61 (88)		23 (92)		0.62
MRS score^e^							
Somatic^f^	4.2 (2.9)	4.0 (2–6)	3.9 (2.9)	3.0 (2–6)	5.1 (2.5)	5.0 (4–7)	**0.033**
Urogenital^f^	2.5 (2.4)	2.0 (1–4)	2.2 (2.3)	2.0 (0–4)	3.2 (2.3)	2.0 (2–5)	0.091
Psychologcal^f^	3.8 (3.0)	3.0 (1–6)	3.9 (3.1)	3.0 (1–6)	3.8 (2.8)	4.0 (2–6)	0.90
Total^f^	10 (6.4)	9.5 (5–15)	9.8 (6.6)	8 (4–15)	12 (5.6)	11 (10–15)	0.068
Depression score^d,g^							
No	58 (53)		36 (52)		12 (48)		
Mild	45 (41)		29 (42)		11 (44)		
Moderate	7 (6.4)		4 (58)		2 (8)		0.54
Major	0 (0.0)		0 (0.0)		0 (0.0)		
Sumscore ^h^	8.2 (5.9)	6.0 (4–12)	8.6 (6.0)	6 (4–13)	7.6 (5.6)	7 (4–11)	0.58

^a^ Still menstruation

^b^ No menstruation since more than one year ago

^c^ First figure mean values (SD)

^d^ *n* (%)

^e^ Subscale and total Menopause Rating Scale (MRS) scoring. The reference values for the symptoms are follow: > 8 (somatic), > 6 (psychological), > 3 (urogenital) and > 16 (total MRS) were defined as severe. First figure mean (SD) second figure median (25th and 75th percentile). Degree of severity of the MRS and its domains indicated; Total score; No, little (0–4), Mild (5–8), Moderate (9–16), Severe (17+), Psychological domain; No, little (0–1), Mild (2–3), Moderate (4–6), Severe (7+), Somatic domain; No, little (0–2), Mild (3–4); Moderate (5–8), Severe (9+), Urogenital domain; No, little (0), Mild (1), Moderate (2–3), Severe (4+)

^f^ *n* 65

^g^ Montgomery-Asberg Depression Rating Scale (MADRS) scoring

^h First^ figure n (%) First figure mean (SD) second figure median (25th and 75th percentile). International standards; 0–6 p no depression, 7–19 p, mild depression, 20–34 p moderate depression, > 34 p severe depression

The MRS score for the somatic sub-scale indicated mild symptoms while the urogenital and psychological sub-scale scores as well as the total score reported moderate symptoms among patients (Tables 1 and 2). Almost every second woman had mild or moderate depression (Table 1).

**Table 2:** Prevalence of menopause symptoms in Menopause Rating Scale (MRS) for Swedish women aged 45–55

Symptoms	None % (*n*)	Mild % (*n*)	Moderate % (*n*)	Severe % (*n*)	Very severe % (*n*)
Somatic^a^					
Hot flushes, sweating:	34 (37/109)	32 (35/109)	29 (32/109)	4.0 (4/109)	1.0 (1/109)
Heart discomfort:	52 (56/108)	28 (30/108)	19 (21/108)	–	1.0 (1/108)
Sleeping problems:	33 (36/108)	26 (28/108)	24 (26/108)	13(14/108)	5.0 (5/108)
Muscle and joint problems:	38 (42/108)	21(23/108)	28 (31/108)	10 (11/108)	2.0 (2/108)
Psychological^a^					
Depressive mood:	33 (36/108)	26 (28/108)	24 (26/108)	13 (14/108)	5.0 (5/108)
Irritability:	41 (44/108)	37 (40/108)	17 (18/108)	6.0 (6/108)	–
Anxiety:	47 (51/108)	33 (36/108)	16 (17/108)	4.0 (4/108)	–
Physical and mental exhaustion:	27 (29/109)	44 (48/109)	19 (21/109)	8.0 (9/109)	2.0 (2/109)
Urogenital^a^					
Sexual problems:	38 (42/109)	33 (36/109)	20 (22/109)	5.0 (5/109)	4.0 (4/109)
Bladder problems:	50 (53/108)	34 (37/108)	12 (13/108)	3.0 (3/108)	2.0 (2/108)
Dryness of vagina:	53 (57/108)	28 (30/108)	12 (13/108)	5.0 (5/108)	3.0 (3/108)

^a^ Higher values indicated more severe symptoms

The most frequently reported MRS symptoms were; physical and mental exhaustion, depressive mood, sleep problems, hot flushes, muscle and joint problems and sexual problems (Table 2).

The most frequently reported depressive symptoms were; concentration difficulties, reduced sleep and inner tension (Table 3).

**Table 3:** Prevalence of depressive symptoms in the Montgomery-Asberg Depression Rating Scale (MADRS) questionnaire among Swedish women aged 45–55 (*n* = 110)

MADRS scoring							
Symptoms^a^	0	1	2	3	4	5	6
Reported sadness:	78 (86)	7.3 (8)	12 (13)	0.91 (1)	1.8 (2)	0	0
Inner tension:	39 (43)	22 (24)	27 (30)	2.7 (3)	9.1 (10)	0	0
Reduced sleep:	37 (41)	12 (13)	27 (30)	8.2 (9)	13 (14)	1.8 (2)	0.91 (1)
Reduced appetite:	74 (81)	19 (21)	5.5 (6)	0.91 (1)	0.91 (1)	0	0
Concentration difficulties:	35 (38)	29 (32)	26 (28)	4.5 (5)	5.5 (6)	0.91 (1)	0
Lassitude:	43 (47)	27 (30)	23 (25)	2.7 (3)	2.7 (3)	1.8 (2)	0
Insensitivity:	45 (49)	29 (32)	28 (24)	3.6 (4)	0.91 (1)	0	0
Pessimism:	42 (46)	21 (23)	24 (26)	4.5 (5)	8.2 (9)	0.91 (1)	0
Suicidal thoughts:	75 (82)	16 (18)	7.3 (8)	0.91 (1)	0.91 (1)	0	0

^a^MADRS scoring 0–6 where higher values indicated more severe symptoms. Data were showing in % (*n*)

**Fig. 1:**
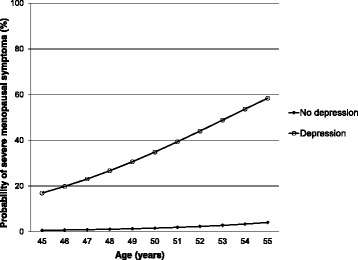
Probability of severe menopausal symptoms among women. Depression = MADRS ≥7 (mild or moderate or severe depression). Severe menopausal symptoms = MRS ≥ 17

Depression and age were associated with more severe symptoms according to MRS (Table 4, Fig. 1). This adjusted model accounted for approximately 39% of the variance of MRS related symptoms (Nagelkerke R^2^) with an AUC (c-statistics) of 0.86 (0.78–0.93, *p* < 0.001).

**Table 4:** Factors associated with menopausal symptoms as measured with Menopause Rating Scale (MRS) (*n* = 109)

	Menopausal symptoms							
	Somatic symptoms >8		Urogenital symptoms >3		Depression symptoms >6		Total of symptoms >16	
	OR (95% CI)		OR (95% CI)	p-value	OR (95% CI)	*p*-value	OR (95% CI)	*p*-value
*Unadjusted*								
Age^a^	1.1 (0.87–1.4)	0.46	1.2 (1.0–1.4)	**0.014**	1.1 (0.96–1.3)	0.15	1.2 (1.01–1.4)	**0.037**
Living with partner	1.0 (0.11–8.8)	1.0	1.8 (0.36–8.6)	0.48	1.3 (0.27–6.5)	0.74	4.3 × 10^8^ (0.0-∞)	1.0
Working^b^	0.11 (0.026–0.47)	**0.0028**	0.55 (0.18–1.7)	0.29	0.21 (0.067–0.62)	**0.0051**	0.24 (0.077–0.74)	**0.013**
Tertiary education^c^	0.50 (0.99–2.5)	0.40	0.44 (0.16–1.2)	0.11	0.83 (0.31–2.2)	0.71	0.75 (0.26–2.1)	0.59
Depression^d^	3.4 × 10^8^ (0.0-∞)	1.0	2.2 (0.91–5.5)	0.079	10 (2.8–37)	**0.00044**	31.6 (4.2–247)	**0.00099**
Menopause^e^	0.42 (0.48–3.7)	0.44	1.3 (0.50–3.8)	0.53	0.61 (0.18–2.03)	0.42	1.1 (0.36–3.7)	0.82
*Adjusted*								
Age^a^							1.2 (1.0–1.5)	**0.041**
Depression^d^					10 (2.8–37)	**0.00044**	33 (4.2–270)	**0.00091**

^a^ Odds Ratio for an increase in age of one year between 45 and 55 years

^b^ Working more than one hour/week

^c^ Primary school/ secondary school vv tertiary school

^d^ Montgomery-Asberg Depression Rating Scale (MADRS) score ≥ 7

^e^ Menopause one year after last menstruation

## Discussion

It appears that depression and increasing age are associated with more severe symptoms in women attending PHC.

### Strengths and weaknesses

The main strength of this study is the use of the validated self-reported questionnaire MRS and linking this to other symptoms in women attending PHC.

The psychological subscale of MRS contains an element of anxiety (item 6) with no equivalent items in MADRS. MADRS contains several items with no equivalent items in MRS. In summary MRS and MADRS overlap significantly but also have important differences. This is further reinforced by the fact that Spearmann’s correlation coefficient between MADRS ≥7 and MRS psychological subscale >6 is only 0.39 (*n* = 108) which does not prohibit performing a regression analysis with these variables.

One weakness is that women were offered participation in a way that made it difficult to estimate the number of non-responders. Furthermore information about co-morbidities was not collected and cannot be adjusted for.

### The association between menopause, depression, age and miscellaneous symptoms

Our study indicated that age and some MRS related symptoms are related to menopause (Table 1). However, this relation is not seen in the multivariate analysis where menopause does not make it to the adjusted model (Table 4). However, this study was not designed to correlate symptoms to menopause but to correlate age, depression and miscellaneous factors to MRS related symptoms irrespective of menopause.

### Mental health, stress and sick leave

This study shows that psychological symptoms frequently reported were physical and mental exhaustion, decrease in concentration, inner tension and anxiety (Tables 2 and 3). Such symptoms may be related to mental stress or other problems around the women [34]. Stress related symptoms or crisis are not necessarily pathological but may imply an unhealthily prolonged psychological burden, reducing opportunities for recovery [34]. Mental illness has increased in the population and become a Swedish public health issue and seems to be more common among women than men [17, 35]. It is also the leading cause of long-term sick leave among women in Sweden [17]. It is shown that early prevention against mental illness and fatigue reduces future sick leave with significant financial gains for the women themselves, employers and the health care system [18, 35, 36]. Hence, it is important to focus on women’s mental health when they visit PHC.

### Sleeping problems

Sleeping problems were the most prevalent reported symptom among women with moderately severe MRS related symptoms (Tables 2 and 3). Sleeping problems can increase the risk of developing mental illhealth and cardiovascular disease, highlighting the importance of sleep for a healthy life [12, 37]. Moreover, increased vulnerability and potentially greater psychological burdens at this age might also cause sleep problems [38]. Due to its high prevalence sleeping problems should be actively explored among women attending PHC for other menopausal symptoms.

### Pain

Musculoskeletal pain is a disorder usually affecting middle aged women [8, 39] and this was also frequently reported in this study (Table 2). Muscle and joint pain is a common cause of sick leave and impaired self-rated health and sleep quality [17]. Furthermore, pain together with mental ill health are common reasons for long-term sick leave in Swedish women. Hence, musculoskeletal symptoms should be actively asked for in women attending PHC for other menopausal symptoms.

### Symptoms from the heart

The incidence of cardiovascular disease (CVD) increases in the age 45–55 years [11, 12, 40]. In accordance with previous research almost half of the women in the present study had heart discomfort (Table 2) with awareness of heart beat, heart skipping, heart racing and tightness [11, 40]. Moreover, this risk may increase with mental illness and sleep problems [37, 38]. Thus, it is important to increase women’s awareness of any cardiovascular risk factors they may have [13, 41].

### Sexuality

This study shows that a majority of women reported sexual problems such as reduced sexual desire, activity and satisfaction (Table 2) of moderate severity (Table 1). Moreover, about half of the women had bladder symptoms and vaginal dryness (Table 2) which is in line with previous studies [8]. A common cause is local oestrogen deficiency resulting in urinary incontinence, vaginal dryness, burning and pain during intercourse, itching and frequent urinary tract infections. All these symptoms may cause sexual problems and decreased sexual desire [42–44]. Consequently, it is important to increase awareness about possible local estrogen deficiency and provide treatment if needed. Other possible causes for reduced sexual desire are miscellaneous changes in women’s daily lives and in relationships.

### Early detection important but difficult

This study confirms previous findings of a high prevalence of mental illness and psychiatric symptoms during menopause [7, 17, 45, 46]. Furthermore, it seems that the prevalence of depression and menopausal symptoms increase with age (Table 4 and Fig. 1). This is a clinically relevant finding since a previous Swedish study showed that long-standing symptoms before a PHC consultation was associated with a prolonged recovery time, underlining the importance of early detection of these symptoms [23, 36, 47]. However, early detection of depression and mental ill health is difficult since one third of depressed women only mention somatic symptoms when visiting PHC [46].

### The transition between 45 and 55 years of age

The transition between 45 and 55 years of age is a natural change in a woman’s life and consequently seen as natural and positive by some, while others see it as unwanted. This period involves profound psychosocial changes such as children leaving home, care for aging parents, changes in marital relationships, and work-related illness [35, 47]. It is important to carefully evaluate symptoms of illness to sort out the true depression from other aspects of mental health illness and crisis reactions [17, 45–47]. The latter can usually be prevented by having healthy social support [35].

A Swedish study revealed that girls in the transition phase (early puberty) should be informed early and before the first menstruation if they are to maintain their positive attitudes towards their body and menstruation [48]. It is possible that early information about health issues in the age group 45–55 is equally relevant for women before this time period occurs. Health education interventions for these women are likely to be beneficial for their knowledge and health [49, 50]. It should be explored in future studies if such preventative counseling of women attending PHC actually results in significantly reduced risk for future illness.

## Conclusion

This study elucidates the diversity of somatic, urogenital and psychological symptoms in the transitional period between 45 and 55 years of age. It appears that mental health is an important aspect to implement in preventive counseling of menopause women in PHC. Future studies should investigate if preventive intervention for women at high risk of MRS related symptoms, according to the nomogram produced in this study, is effective.

## Abbreviations

APN: Advanced practice Nurse
AUC: Area under curve
CVD: Cardiovascular disease
DN: District nurse
MADRS: Montgomery Åsberg depression rating scale
MRS: Menopause rating scale
OR: Odds ratio
PHC: Primary health care
SD: Standard deviation
